# Risk factors for illness severity in patients with COVID-19 pneumonia: a prospective cohort study

**DOI:** 10.7150/ijms.51205

**Published:** 2021-01-01

**Authors:** Nannan Zhang, Hairong Zhang, Yanhua Tang, Hao Zhang, Aiying Ma, Fang Xu, Yu Sun, Luning Jiang, Fangzhen Shan

**Affiliations:** 1 Department of Pulmonary and Critical Care Medicine, Affiliated Hospital of Jining Medical University, Jining 272029, Shandong, China.; 2 Department of surgery, Jining Infectious Disease Hospital, Jining City 272000, Shandong, China.; 3 Department of hematology, Affiliated Hospital of Jining Medical University, Jining 272029, Shandong, China.; 4 Department of Intensive care unit II, Affiliated Hospital of Jining Medical University, Jining 272029, Shandong, China.; 5 Department of infectious disease, Jining Infectious Disease Hospital, Jining City 272000, Shandong, China.; 6 Medical Research Center, Affiliated Hospital of Jining Medical University, Jining 272029, Shandong, China.; 7 Hefei National Laboratory for Physical Science at Microscale and School of Life Science, University of Science and Technology of China, Hefei 230026, Anhui, China.

**Keywords:** SARS-CoV-2, COVID-19, severity, risk factor, prospective studies

## Abstract

**Background:** Although COVID-19 pneumonia is spreading internationally, knowledge regarding the factors associated with the illness severity of patients remains limited. We aimed to identify the factors associated with the disease severity of patients with COVID-19 pneumonia induced by a novel coronavirus, severe acute respiratory syndrome coronavirus 2 (SARS-CoV-2).

**Methods:** We prospectively enrolled a single-center case series of adult patients with COVID-19 admitted to the Infectious Disease Hospital of Jining, Jining City, Shandong Province, China, from January 24 to March 1, 2020. Demographics, clinical characteristics, and laboratory findings were compared to investigate the risk factors related with the disease severity of COVID-19 pneumonia patients.

**Results:** We included a total of 78 patients with COVID-19 pneumonia, of whom 6 had the severe type. As compared to a moderately ill cohort, our analysis showed that shortness of breath, fatigue, longer days from illness onset to diagnosis confirmed, neutrophil percentages > 70%, neutrophil counts > 6.3 × 10^9^/L, lymphocyte percentages < 20%, lymphocyte counts < 1.0 × 10^9^/L, platelet < 100 × 10^9^/L, C-reactive protein (CRP) > 10 mg/L, neutrophil to platelet ratio (NPR) > 2.3, neutrophil to lymphocyte ratio (NLR) > 3.9, aspartate aminotransferase (AST) > 40 U/L, albumin < 40 g/L, lactate dehydrogenase (LDH) > 245 U/L, and glucose > 6.1 mmol/L were predictors of disease severity in COVID-19 pneumonia. In the sex-, age-, and comorbid illness-matched case-control study, neutrophil percentages > 70%, neutrophil counts > 6.3 × 10^9^/L, lymphocyte percentages < 20%, NPR > 2.3, NLR > 3.9, albumin < 40 g/L, and LDH > 245 U/L remained associated with the early detection and identification of severe patients.

**Conclusion:** We demonstrated that neutrophil percentages > 70%, neutrophil counts > 6.3 × 10^9^/L, lymphocyte percentages < 20%, NPR > 2.3, NLR > 3.9, albumin < 40 g/L, and LDH > 245 U/L might predict the severity of illness in patients with COVID-19 pneumonia.

## Background

COVID-19 pneumonia has become a worldwide pandemic since it first emerged in December, 2019, in Wuhan City, Hubei Province, China 1-3. The severe acute respiratory syndrome coronavirus 2 (SARS-CoV-2), belonging to the β-coronavirus family, is similar to some known coronaviruses, such as SARS-CoV and middle east respiratory syndrome (MERS)-CoV, in terms of its surface spike proteins 4, 5. As at September 15, 2020, the COVID-19 pneumonia pandemic has been reported in 188 countries globally, with 29,316,738 confirmed cases and 929,236 deaths 6. COVID-19 has been declared a public health emergency by the World Health Organization, threatening global health and economies. Unfortunately, the COVID-19 pneumonia pandemic is still ongoing and not well controlled at present.

Emerging studies have reported the epidemiology and clinical characteristics of COVID-19. Symptoms of SARS-CoV-2 infection range from none to critical illness, and often present as fatal acute respiratory distress syndrome 7-9. In one published study including 99 patients with COVID-19 pneumonia from Wuhan Jinyintan Hospital, 17% patients developed severe symptoms 10. The percentage of severe cases was 11.3% in another cohort of hospitalized patients with COVID-19 pneumonia 11. Moreover, in a study in the New York City Area from America, a case series involved 5700 patients, the percentage of severe cases were 14.2% 12. Though results from these three studies have indicated that older age, and multiple pulmonary lobes involvement and pleural effusion were associated with the illness severity, factors related with the severity of illness are still poorly understood 13. Initial studies were mostly retrospectively; whereas, prospective studies on the prediction of disease severity are limited 14-17.

In the present study, a total of 78 adult patients confirmed with COVID-19 infection hospitalized at the Institute of Infectious Diseases, Jining Hospital, which was a COVID-19-designated hospital, between January 24 and March 1, 2020 were recruited. The patients included 72 moderate and 6 severe cases, on the basis of The Diagnosis and Treatment of COVID-19 guidelines (7th version) published by the National Health Commission of China 18. We compared the differences in clinical characteristics and laboratory findings between moderate and severe cases, in order to identify the clinical and laboratory parameters related to illness severity of patients with COVID-19 pneumonia. It was identified that if neutrophil percentages > 70%, neutrophil counts > 6.3 × 10^9^/L, lymphocyte percentages < 20%, NPR > 2.3, NLR > 3.9, albumin < 40 g/L, and LDH > 245 U/L might be risk factors for disease severity among patients with COVID-19 pneumonia. Our study will provide a supplement to the researches on epidemic of COVID-19 pneumonia, and references for its diagnosis.

## Methods

### Study patients and data collection

Clinical characteristics and laboratory findings were prospectively collected from a single-center cohort of 78 consecutive adult patients confirmed with COVID-19 pneumonia who were admitted to Jining Infectious Disease Hospital, Jining City, Shandong Province, China, from southeast Shandong province, between January 24 and March 1, 2020. All patients were diagnosed with COVID-19 through a real-time reverse transcriptase-polymerase chain reaction assay using oropharyngeal swab specimens from the upper respiratory tract. We divided the disease severity into a moderate or severe group based on the latest Chinese COVID-19 Management Guidance (7th edition) 18. Our study was approved by the Ethics Committee of the Jining Infectious Disease Hospital, Jining City, Shandong Province, China (No. 20200102). All methods were performed in accordance with the relevant guidelines and regulations. Each patient provided informed consent at the time we started to collect and analyze the data.

Medical information of each patient including demographic data, clinical characteristics, laboratory results, and outcomes was extracted prospectively through review of electronic medical records. All the data were checked and reviewed by two doctors from an affiliated Hospital of Jining Medical University, independently.

### Statistics

All statistical tests were performed using SPSS version 23.0 (SPSS Inc., Chicago, IL, USA). Continuous variables are presented as means ± standard deviations (SD) or median with interquartile range (IQR), and were compared using either the Student's t-test for normally distributed variables or the Mann-Whitney test for non-normally distributed variables, as appropriate. Continuous variables were tested for normality using the Shapiro-Wilk test. Differences between categorical variables were determined using Pearson's Chi-squared test or Fisher's Exact Test, as appropriate, and presented as frequencies and percentages (%). Statistical analyses were two-sided and significance was set at *P* < 0.05.

## Results

### Clinical characteristics on admission

A cohort of 78 patients who were hospitalized at Jining Infectious Disease Hospital, Jining City, Shandong Province, China, between January 24 and March 1, 2020 were recruited. All patients were diagnosed with COVID-19 pneumonia by a positive SARS-CoV-2 nucleic acid test. As shown in **Table 1**, the median age of patients was 43.82 ± 15.91 years, and males accounted for 76.9%. Of 78 patients, 6 (7.7%) cases developed into a severe type. As for comorbidities, there were no significant differences in the percentages of hypertension, diabetes, cardiovascular disease, tumor, cerebrovascular disease, and lung disease between the moderate and severe cohorts (all *P* > 0.05). We surveyed smoking habits and alcohol consumption in all patients. No significant differences were found in term of the sex percentage, coexisting disorders, smoking, or alcohol consumption between these two groups (all *P* > 0.05).

Among the clinical symptoms including fever, shortness of breath, dry cough, fatigue, sputum production, gastrointestinal symptoms, hemoptysis, myalgia, headache, pharyngodynia, and rhinobyon, the top five common symptoms were fever (80.8%), followed by dry cough (46.2%), gastrointestinal symptoms (17.9%), fatigue (14.1%), and shortness of breath (10.3%) in the total patient population. The mean age of the severe group was not different from that of the moderate cohort. We noted that shortness of breath and fatigue appeared more frequently in the severe cohort than in the moderate cohort (50% vs. 6.9%, *P* = 0.013; 50% vs. 11.1%, *P* = 0.034). Furthermore, the days from illness onset to diagnosis confirmed in severe group were significantly longer than that in moderate group [9.0 (6.0-16.0 vs. 5.0 (3.0-7.0),* P* =0.042].

### Laboratory data

As compared to patients with moderate disease, the severe patients had a higher level of white blood cells (WBC) [8.35 (5.73-13.15) vs. 5.16 (4.15-6.46) × 10^9^/L, *P* = 0.008], neutrophil percentages [86.95 (76.9-91.88)% vs. 55.2 (48.08-65.35)%, *P* < 0.001], neutrophil counts [6.47 (5.1-11.09) vs. 2.84 (2.08-3.88) × 10^9^/L, *P* = 0.001], and C-reactive protein (CRP) [58.08 (19.26-90.37) vs. 0.54 (0.50-4.06) mg/L, *P* < 0.001], but lower levels of lymphocyte percentages [9.0 (4.08-13.38)% vs. 31.65 (22.6-39.95)%, *P* < 0.001], lymphocyte counts [0.74 (0.42-1.31) vs. 1.57 (1.18-1.89) × 10^9^/L, *P* = 0.002], and platelets [185 (79-230.75) vs. 243.75 (195.0-308.75) × 10^9^/L, *P* = 0.022] (**Table 2**). According to the parameters from the peripheral blood routine tests, we then calculated the neutrophil to platelet ratio (NPR), neutrophil to lymphocyte ratio (NLR), and platelet to lymphocyte ratio (PLR), in order to further investigate the factors related with illness severity between the mild and severe groups. Notably, indices including NPR [4.64 (2.40-11.44) vs. 1.24 (0.88-1.67), *P* < 0.001] and NLR [9.79 (5.87-22.96) vs. 1.72 (1.17-2.77), *P* < 0.001] were significantly increased in severe patients as compared to those in moderate cases.

Compared to the patients in the moderate group, severe patients were more susceptible to hepatic insufficiency, as indicated by the elevation of aspartate aminotransferase (AST) [33.5 (23.75-68) vs. 21 (18-27) U/L, *P* = 0.004] and lactate dehydrogenase (LDH) [389.5 (241.5-497.5) vs. 197.5 (160.25-229.75) U/L, *P* = 0.002], and the decreased albumin concentration [35.5 (32.75-38.25) vs. 42 (39-44) g/L, *P* = 0.003]. In addition, severe patients also showed more frequently disturbed blood electrolytes and impaired glucose, as demonstrated by the reduction of sodium [136 (134.25-137.5) vs. 141 (139-143) mmol/L, *P* = 0.001] and calcium [96.5 (93.75-98) vs. 100 (97-102) mmol/L, *P* = 0.015], and the increased glucose level [6.2 (5.63-11.5) vs. 4.9 (4.4-5.6) mmol/L, *P* = 0.007] (**Table 2**).

### Factors associated with disease severity

For all the demographic data, clinical characteristics, and laboratory data, our analyses revealed that the severe group had longer days from illness onset to diagnosis confirmed, and significant higher proportions of patients showing shortness of breath, fatigue, neutrophil percentages > 70%, neutrophil counts > 6.3 × 10^9^/L, lymphocyte percentages < 20%, lymphocyte counts < 1.0 × 10^9^/L, platelet < 100×10^9^/L, CRP > 10 mg/L, NPR > 2.3, NLR > 3.9, AST > 40 U/L, albumin < 40 g/L, LDH > 245 U/L, and glucose > 6.1 mmol/L than that the moderate cohort (**Table 1, Table 2**). Therefore, our findings suggest that these factors might be associated with the disease severity of patients with COVID-19 pneumonia.

To further investigate the risk factors related with illness severity of COVID-19 pneumonia patients, we performed a case-control study at a ratio of 2:1. We selected 12 sex-, age- and underlying comorbidity-matched patients from the moderate patient group and matched them with 6 severe cases. The ages of the moderate patients were the same or ± 2 years as matched to each severe case, as many of the moderate patients were younger. As shown in **Table 3**, we found no significant differences in the demography and clinical presentation between severe patients and the matched case-control severe cases. In addition, there were also no significant differences in the days from illness onset to admission, days from illness onset to diagnosis confirmed, and days from diagnosis confirmed to virus turned negative. Contrary to the moderate patients, there were significantly elevated concentrations of WBC, neutrophil percentages, neutrophil counts, CRP, and LDH in severe cases; while there were reduced levels of lymphocyte percentages, lymphocyte counts, NPR, NLR, albumin, and sodium in severe patients (**Table 4**). **Table 3** and **Table 4** show that the matched case-control severe cases had higher percentages of neutrophil percentages > 70%, neutrophil counts > 6.3 × 10^9^/L, lymphocyte percentages < 20%, NPR > 2.3, NLR > 3.9, albumin < 40 g/L, and LDH > 245 U/L than those in the moderate cohorts.

## Discussion

The global number of cases of SARS-CoV-2 infection has continued to rise since the start of the outbreak. Currently, SARS-CoV-2 infection has resulted in more than 80,000 laboratory-confirmed cases in China, with an overall mortality of appropriately 5% 19. Unfortunately, despite many clinical drug trials underway, such as those for remdesivir, choloroquine, or lopinavir/ritonavir, no effective licensed drugs specifically targeting SARS-CoV-2 are currently available 20-22. The genomic sequence of SARS-CoV-2, published in 2020, shares 82% similarity with that of SARS-CoV 4, 23. Although similar to the severe acute respiratory syndrome (SARS) that originated in Guangdong province in 2003, SARS-CoV-2 infection can develop rapidly into respiratory failure and death 24, 25. However, knowledge of risk factors associated with the illness severity of patients remains limited. In the present study, we summarized the epidemiological characteristics and clinical features of cases of COVID-19 in Southwest Shandong province. COVID-19 patients were divided into a moderate cohort and a severe cohort, and we identified that neutrophil percentages > 70%, neutrophil counts > 6.3 × 10^9^/L, lymphocyte percentages < 20%, NPR > 2.3, NLR > 3.9, albumin < 40 g/L, and LDH > 245U/L might be risk factors for disease severity among COVID-19 pneumonia patients.

As for systemic organ indices, our results revealed that the severe COVID-19 group presented relatively elevated WBC counts, neutrophil percentages, and neutrophil counts; high CRP levels; and decreased lymphocyte percentages, lymphocyte counts, and platelet counts. Moreover, patients in the severe cohort displayed a higher prevalence of neutrophil percentages > 70%, neutrophil counts > 6.3 × 10^9^/L, lymphocyte percentages < 20%, lymphocyte counts < 1.0 × 10^9^/L, platelet < 100 × 10^9^/L, CRP > 10 mg/L than those in the moderate group, indicating that these parameters might be risk factors associated with COVID-19 severity in the general population. These findings are consistent with other studies that have reported abnormalities in hematological parameters including neutrophilia, lymphocytopenia, and thrombocytopenia in association with disease severity or mortality for patients with COVID-19 pneumonia 26, 27. Elevated CRP concentrations have been reported to be connected with poor outcomes and death, and more severe cases displayed more marked elevation as compared with moderate cases 26. These laboratory data might suggest that patients with COVID-19 have impaired hematological and cellular immune systems, as reported previously 28. In our present analysis, neutrophil percentages > 70%, neutrophil counts > 6.3 × 10^9^/L, and lymphocyte percentages < 20% remained in the sex-, age-, and comorbid illness-matched control cases. Studies involving more patients are necessary to further consolidate our preliminary conclusions. Furthermore, the mechanisms of cellular immune system dysfunction in COVID-19 deserve further investigation.

A previous study reported that infection with SARS-CoV-2 could cause the production of a range of systemic cytokines and chemokines, such as interleukin (IL)-2, IL-6, IL-7, IL-10, tumor necrosis factor-α, monocyte chemoattractant protein-1 and these were significantly elevated in severe patients with COVID-19 pneumonia compared with moderate cases, leading to intense systemic inflammation 29-31. A meta-analysis recently reported that thrombocytopenia was associated with illness severity among COVID-19 patients, and a dramatic drop in platelet counts in deceased patients 32. Interestingly, another study reported that peak platelet count during treatment, but not platelet count at baseline, was associated with a poorer prognosis; and PLR at peak platelet counts was an independent influencing factor in severe patients evaluated by multivariate analysis, thus suggesting a degree of cytokine storm due to pronounced platelet activation 33. In contrast, we found that platelet counts were greatly reduced at admission in the severe group compared with the moderate group, while no significant difference was discovered with regard to PLR between the two groups, demonstrating that patients infected with SARS-CoV-2 might have microcirculatory dysfunction. We suggest that drugs should be administered early enough to inhibit platelet activation and prevent thrombolysis. Further data are necessary to further investigate the role of platelets in COVID-19 pneumonia.

NPR, NLR, platelet to lymphocyte ratio (PLR), and lymphocyte to monocyte ratios have been widely studied as potential predictors of systemic inflammatory responses 34, 35. Notably, one important finding in our results was that NPR and NLR, while not PLR, were remarkably increased in severe patients as compared to either the moderate patients or the sex-, age-, and comorbid illness-matched control cases. Besides, we defined NPR at 2.3 and NLR at 3.9 using the receiver operation characteristic curve. Moreover, NPR > 2.3 and NLR > 3.9 were risk factors for severity of patients with COVID-19 pneumonia. Our results were in line with prior studies from a cohort of patients with COVID-19 indicating that NLR is a risk factor for poor clinical outcomes or mortality 36, 37. Therefore, these findings suggest that unbalanced immunity showing exuberant activation of the inflammatory system and inadequate response of the adaptive immune system might lead to serious illness.

In our study, we demonstrated that severe patients were susceptible to hepatic insufficiency, which is in accordance with previous reports 38-40. The laboratory indices reflecting derangement of liver function that were associated with disease severity of COVID-19 pneumonia were increased AST and LDH, and reduced albumin. In addition, severe cases presented internal environment disturbance, as the laboratory parameters reflecting internal environment disturbance that related with severe illness were decreased sodium and calcium, and elevated glucose levels compared with the general population. However, there were no significant differences in the levels of potassium and sodium between critical and non-critical patients as previously reported 29. Most importantly, LDH and albumin parameters remained valid in the sex-, age-, and underlying illness-matched control data. Therefore, more attention should be urgently paid to organ protection strategies, especially liver protection, and maintenance of internal environmental balance during the course of illness in severe COVID-19 patients.

Unlike most other retrospective studies reported previously 41-44, we conducted a perspective study in order to characterize the risk factors associated with the severity of illness among COVID-19 patients. Some prospective reports have implicated several factors related with the critical disease and mortality for patients afflicted with COVID-19; however, prospective studies of disease severity are limited 45, 46. Reported results have demonstrated that older age, comorbidities, obesity, and laboratory abnormalities including impairment of oxygen, elevated CRP concentration, higher D-dimer level, and increased serum ferritin were associated with severe illness or mortality 14-17. Using the continued admitted patients diagnosed with COVID-19 prospectively, we have suggested that neutrophil percentages > 70%, neutrophil counts > 6.3 × 10^9^/L, lymphocyte percentages < 20%, NPR > 2.3, NLR > 3.9, albumin < 40 g/L, and LDH > 245 U/L were risk factors for illness severity in COVID-19 patients. Our study also featured a case-control study at 2:1 that further validated the findings, which were different from the prospective reports. In contrast with some studies previously 14, 15, not only did we identify some factors for illness severity, but we also further provided the specific values of these indicators in COVID-19. Therefore, our study might to some extent add some knowledge to the predictors for disease severity among COVID-19 patients.

The limitations should be addressed in further research. Due to the single-center study design and limited number of cases, caution should be applied, as some of these conclusions are preliminary. In addition, another note of caution is due to the limited number of severe cases. Since there were only six patients, it is difficult to evaluate risk factors for illness severity using univariate or multivariate methods. Moreover, since the pathogen of SARS-CoV-2 was identified, the dynamics of viral load and antibody titer are unclear. These need to be validated in following studies.

## Conclusions

In conclusion, we identified many factors, such as shortness of breath, fatigue, longer days from illness onset to diagnosis confirmed, neutrophil percentages > 70%, neutrophil counts > 6.3 × 10^9^/L, lymphocyte percentages < 20%, lymphocyte counts < 1.0 × 10^9^/L, platelet < 100 × 10^9^/L, CRP > 10 mg/L, NPR > 2.3, NLR > 3.9, AST > 40 U/L, albumin < 40 g/L, LDH > 245 U/L, and glucose > 6.1 mmol/L related to disease severity among COVID-19 pneumonia patients. In the sex-, age-, and comorbid illness-matched case-control study, we further revealed that neutrophil percentages > 70%, neutrophil counts > 6.3 × 10^9^/L, lymphocyte percentages < 20%, NPR > 2.3, NLR > 3.9, albumin < 40 g/L, and LDH > 245 U/L remained relative risk factors for illness severity in COVID-19 pneumonia patients with similar ages and underlying diseases.

## Figures and Tables

**Table 1:** Summary of patient clinical characteristics.

Characteristics	Total (n = 78)	Moderate (n =72)	Severe (n = 6)	*P* value
Age (years)	43.82±15.91	43.47±16.00	48.00±15.49	0.507
Gender, male, n (%)	60 (76.92)	45 (62.50)	5 (83.33)	0.562
Coexisting diseases, n (%)				
Hypertension	11 (14.10)	10 (13.89)	1 (16.67)	1.000
Diabetes	11 (14.10)	10 (13.89)	1 (16.67)	1.000
Cardiovascular disease	8 (10.26)	8 (11.11)	0	1.000
Tumor	2 (2.56)	2 (2.78)	0	1.000
Cerebrovascular disease	3 (3.85)	3 (4.17)	0	1.000
Smoking, n (%)	7 (8.97)	6 (8.33)	1 (16.67)	0.442
Alcohol consumption, n (%)	8 (10.26)	7 (9.72)	1 (16.67)	0.490
Symptoms, n (%)				
Fever	63 (80.77)	57 (79.17)	6 (100.00)	0.481
Shortness of breath	8 (10.26)	5 (6.94)	3 (50.00)	0.013
Dry Cough	36 (46.15)	33 (45.83)	3 (50.00)	1.000
Fatigue	11 (14.10)	8 (11.11)	3 (50.00)	0.034
Sputum production	6 (7.69)	6 (8.33)	0	1.000
Gastrointestinal symptoms	14 (17.95)	12 (16.67)	2 (33.33)	0.639
Hemoptysis	1 (1.28)	0	1 (16.67)	0.077
Myalgia	4 (5.13)	4 (5.56)	0	1.000
Headache	3 (3.85)	3 (4.17)	0	1.000
Pharyngodynia	7 (8.97)	7 (9.72)	0	1.000
Rhinobyon	3 (3.85)	3 (4.17)	0	1.000
Days from illness onset to admission, median days (IQR)	6.5 (4.0-11.25)	6.0 (4.0-11.75)	8.5 (3.0-12.25)	0.903
Days from illness onset to diagnosis confirmed, median days (IQR)	5.0 (3.0-8.0)	5.0 (3.0-7.0)	9.0 (6.0-16.0)	0.042
Days from diagnosis confirmed to virus turned negative, median days (IQR)	11.0 (8.0-15.75)	11.0 (8.0-16.0)	8.0 (7.0-12.5)	0.240

Variables are presented as mean ± standard deviation (SD). Other values are presented as numbers (%). *P* < 0.05 was considered statistically significant.

**Table 2:** Laboratory features of 78 COVID-19 patients.

Characteristic	Total (n = 78)	Moderate (n =72)	Severe (n = 6)	*P* value
White blood cells (× 10^9^/L)	5.34 (4.26-6.79)	5.16 (4.15-6.46)	8.35 (5.73-13.15)	0.008
> 10 × 10^9^/L, n (%)	6 (7.69)	4 (5.56)	2 (33.33)	0.065
Neutrophils%	58.95 (48.60-69.93)	55.20 (48.08-65.35)	86.95 (76.90-91.88)	< 0.001
> 70	18 (23.08)	13 (18.06)	5 (83.33)	0.002
Neutrophils (× 10^9^/L)	2.93 (2.19-4.26)	2.84 (2.08-3.88)	6.47 (5.10-11.09)	0.001
> 6.3	10 (12.82)	7 (9.72)	3 (50.00)	0.025
Lymphocytes%	30.85 (20.98-38.45)	31.65 (22.60-39.95)	9.0 (4.08-13.38)	< 0.001
< 20	17 (21.79)	11 (15.28)	6 (100.00)	< 0.001
Lymphocytes (× 10^9^/L)	1.52 (1.12-1.85)	1.57 (1.18-1.89)	0.74 (0.42-1.31)	0.002
< 1.0 × 10^9^/L, n (%)	14 (17.95)	10 (13.89)	4 (66.67)	0.007
Platelet (× 10^9^/L)	234.00 (192.50-302.00)	243.75 (195.00-308.75)	185.00 (79.00-230.75)	0.022
< 100	3 (3.85)	1 (1.39)	2 (33.33)	0.014
C-reactive protein (mg/L)	0.87 (0.50-6.63)	0.54 (0.50-4.06)	58.08 (19.26-90.37)	< 0.001
> 10	13 (16.67)	8 (11.11)	5 (83.33)	< 0.001
Neutrophil to platelet ratio	1.28 (0.91-2.07)	1.24 (0.88-1.67)	4.64 (2.40-11.44)	< 0.001
> 2.3	17 (21.79)	11 (15.28)	6 (100.00)	< 0.001
Platelet to lymphocyte ratio	158.64 (121.20-216.12)	156.84 (120.79-204.17)	250.79 (103.46-492.44)	0.294
Neutrophil to lymphocyte ratio	1.87 (1.20-3.21)	1.72 (1.17-2.77)	9.79 (5.87-22.96)	< 0.001
> 3.9	15 (19.23)	9 (12.50)	6 (100.00)	< 0.001
ALT (U/L)	22.00 (16.00-30.25)	22.00 (16.00-29.50)	23.00 (18.00-84.75)	0.329
AST (U/L)	21.50 (18.00-28.00)	21 (18.00-27.00)	33.5 (23.75-68.00)	0.004
> 40	3 (3.85)	1 (1.39)	2 (33.33)	0.014
Total bilirubin (μmol/L)	10.75 (7.18-13.62)	11.00 (7.33-13.66)	9.63 (6.95-12.25)	0.435
Direct bilirubin (μmol/L)	3.24 (1.95-4.24)	3.23 (1.74-4.31)	3.27 (2.23-3.83)	0.879
Albumin (g/L)	42.00 (38.00-44.00)	42.00 (39.00-44.00)	35.50 (32.75-38.25)	0.003
< 40	28 (35.90)	22 (30.56)	6 (100.00)	0.003
Creatinine (μmol/L)	56.50 (47.00-68.00)	56.50 (47.00-68.00)	58.50 (50.25-67.25)	0.680
Urea (mmol/L)	4.09 (3.28-4.94)	4.10 (3.27-4.90)	3.91 (3.22-5.77)	0.817
LDH (U/L)	203.50 (161.00-245.00)	197.50 (160.25-229.75)	389.50 (241.50-497.50)	0.002
> 245	19 (24.36)	14 (19.44)	5 (83.33)	0.003
Glucose (mmol/L)	5.05 (4.48-5.93)	4.90 (4.40-5.60)	6.20 (5.63-11.50)	0.007
> 6.1	16 (20.51)	12 (16.67)	4 (66.67)	0.017
CK-MB (U/L)	16 (13.00-23.75)	16.50 (13.00-26.00)	15.50 (11.50-17.50)	0.264
Sodium (mmol/L)	141.00 (138.75-143.00)	141.00 (139.00-143.00)	136.00 (134.25-137.50)	0.001
< 135	5 (6.41)	4 (5.56)	1 (16.67)	0.337
Potassium (mmol/L)	4.10 (3.70-4.60)	4.10 (3.70-4.60)	3.65 (3.33-4.50)	0.670
Calcium (mmol/L)	100.00 (96.00-102.00)	100.00 (97.00-102.00)	96.50 (93.75-98.00)	0.015
< 96	13 (16.67)	11 (15.28)	2 (33.33)	0.569

Continuous variables are presented as median with interquartile range (IQR; 25%, 75%) or mean ± standard deviation (SD), as appropriate. Other values are presented as numbers (%). *P* < 0.05 was considered statistically significant. ALT, Alanine aminotransferase; AST, Aspartate aminotransferase; LDH, lactate dehydrogenase; CKMB, Creatine kinase-MB.

**Table 3:** Comparison of clinical characteristics between severe cases and sex-, age-, and underlying disease-matched case-control moderate cases.

Characteristics	Total (n = 18)	Moderate (n =12)	Severe (n = 6)	*P* value
Age (years)	42.78±17.45	47.75±4.26	48.00±6.33	0.974
Gender, male, n (%)	15 (83.33)	10 (83.33)	5 (83.33)	1.000
Coexisting diseases, n (%)				
Hypertension	4 (22.22)	3 (25.00)	1 (16.67)	1.000
Diabetes	3 (16.67)	2 (16.67)	1 (16.67)	1.000
Cardiovascular disease	1 (5.56)	1 (8.33)	0	1.000
Tumor	1 (5.56)	1 (8.33)	0	1.000
Cerebrovascular disease	1 (5.56)	1 (8.33)	0	1.000
Lung diseases^a^	1 (5.56)	0	1 (16.67)	0.333
Smoking, n (%)	2 (11.11)	1 (8.33)	1 (16.67)	1.000
Alcohol consumption, n (%)	2 (11.11)	1 (8.33)	1 (16.67)	1.000
Symptoms, n (%)				
Fever	17 (94.44)	11 (91.67)	6 (100.00)	1.000
Shortness of breath	5 (27.78)	2 (16.67)	3 (50.00)	0.352
Dry Cough	8 (44.44)	5 (41.67)	3 (50.00)	1.000
Sputum production	2 (11.11)	2 (16.67)	0	0.529
Gastrointestinal symptoms	5 (27.78)	3 (25.00)	2 (33.33)	1.000
Hemoptysis	1 (5.56)	0	1 (16.67)	0.333
Pharyngodynia	1 (5.56)	1 (8.33)	0	1.000
Days from illness onset to admission, median days (IQR)	6.0 (4.0-9.0)	5.5 (3.25-7.5)	9.0 (6.0-15.5)	0.130
Days from illness onset to diagnosis confirmed, median days (IQR)	6.0 (4.0-9.0)	6.0 (4.0-8.5)	9.0 (6.0-16.0)	0.195
Days from diagnosis confirmed to virus turned negative, median days (IQR)	9.0 (7.5-15.0)	9.5 (8.0-15.0)	8.0 (7.0-12.5)	0.506

Variables are presented as mean ± standard deviation (SD). Other values are presented as numbers (%). *P* < 0.05 was considered statistically significant.

**Table 4:** Laboratory features of severe cases and age-sex matched case-control moderately ill patients.

Characteristic	Total (n = 18)	Moderate (n =12)	Severe (n = 6)	*P* value
White blood cells (× 10^9^/L)	5.97 (3.97-7.45)	4.83 (3.37-6.39)	8.35 (5.73-13.15)	0.010
>10 × 10^9^/L, n (%)	2 (11.11)	0	2 (33.33)	0.098
Neutrophils%	62.90 (48.45-80.80)	49.80 (47.10-63.45)	86.95 (76.90-91.88)	< 0.001
> 70	6 (33.33)	1 (8.33)	5 (83.33)	0.004
Neutrophils (× 10^9^/L)	3.29 (2.31-5.51)	2.72 (1.74-3.35)	6.47 (5.10-1.09)	< 0.001
> 6.3	3 (16.67)	0	3 (50.00)	0.025
Lymphocytes%	25.25 (11.70-39.53)	34.35 (23.83-41.78)	9.00 (4.08-13.38)	< 0.001
< 20	6 (33.33)	0	6 (100.00)	< 0.001
Lymphocytes (× 10^9^/L)	1.28 (0.77-1.61)	1.52 (1.12-1.87)	0.74 (0.42-1.31)	0.013
<1.0 × 10^9^/L, n (%)	6 (33.33)	2 (16.67)	4 (66.67)	0.107
Platelet (× 10^9^/L)	220.50 (180.75-290.50)	238.50 (209.25-339.25)	185.00 (79.00-230.75)	0.053
< 100	3 (16.67)	1 (8.33)	2 (33.33)	0.245
C-reactive protein (mg/L)	11.88 (1.19-31.24)	2.04 (0.50-14.81)	58.08 (19.26-90.37)	0.002
> 10	9 (50.00)	4 (33.33)	5 (83.33)	0.131
Neutrophil to platelet ratio	1.32 (0.87-2.93)	0.95 (0.60-1.37)	4.64 (2.40-11.44)	< 0.001
> 2.3	7 (38.89)	1 (8.33)	6 (100.00)	< 0.001
Platelet to lymphocyte ratio	167.21 (124.55-299.95)	157.58 (121.95-225.02)	250.79 (103.46-492.44)	0.335
Neutrophil to lymphocyte ratio	2.47 (1.19-6.98)	1.48 (1.16-2.60)	9.79 (5.87-22.96)	< 0.001
> 3.9	6 (33.33)	0	6 (100.00)	< 0.001
ALT (U/L)	24.00 (18.25-49.50)	24.00 (17.00-41.75)	23.00 (18.00-84.75)	0.892
AST (U/L)	25.50 (22.00-36.00)	24.50 (21.25-29.25)	33.50 (23.75-68.00)	0.125
> 40	3 (16.67)	1 (8.33)	2 (33.33)	0.245
Total bilirubin (μmol/L)	10.38 (8.53-13.70)	10.75 (9.00-13.90)	9.63 (6.95-12.25)	0.437
Direct bilirubin (μmol/L)	3.27 (2.25-4.80)	3.10 (2.18-5.00)	3.27 (2.25-3.83)	0.494
Albumin (g/L)	38.00 (35.00-41.25)	40.00 (36.50-42.75)	35.50 (32.75-38.25)	0.024
< 40	11 (61.11)	5 (41.67)	6 (100.00)	0.038
Creatinine (μmol/L)	60.50 (50.50-69.50)	60.50 (51.00-74.75)	58.50 (50.25-67.25)	0.616
Urea (mmol/L)	3.92 (3.23-4.78)	3.92 (3.13-4.67)	3.91 (3.22-5.77)	0.616
LDH (U/L)	236.50 (200.50-353.25)	223.00 (174.25-262.00)	389.50 (241.50-497.50)	0.041
> 245	8 (44.44)	3 (25.00)	5 (83.33)	0.043
Glucose (mmol/L)	5.70 (4.35-9.23)	4.95 (4.13-8.28)	6.20 (5.63-11.50)	0.151
> 6.1	8 (44.44)	4 (33.33)	4 (66.67)	0.321
CK-MB (U/L)	16.00 (11.75-19.50)	16 (11.75-24.75)	15.50 (11.50-17.50)	0.553
Sodium (mmol/L)	139.00 (135.00-141.25)	140.50 (137.50-142.00)	136.00 (134.25-137.50)	0.032
< 135	2 (11.11)	1 (8.33)	1 (16.67)	1.000
Potassium (mmol/L)	4.10 (3.70-4.83)	4.30 (3.85-4.88)	3.65 (3.33-4.50)	0.250
Calcium (mmol/L)	97.50 (95.00-100.00)	99.00 (95.25-100.75)	96.5 (93.75-98.00)	0.213
< 96	5 (27.78)	3 (25.00)	2 (33.33)	1.000

Continuous variables are presented as median with interquartile range (IQR; 25%, 75%) or mean ± standard deviation (SD), as appropriate. Other values are presented as numbers (%). *P* < 0.05 was considered statistically significant.

## Abbreviations

AST: aspartate aminotransferase
CRP: C-reactive protein
LDH: lactate dehydrogenase
IL: interleukin
IQR: median with interquartile range
MERS: middle east respiratory syndrome
NLR: neutrophil to lymphocyte ratio
NPR: neutrophil to platelet ratio
PLR: platelet to lymphocyte ratio
SARS: severe acute respiratory syndrome
SARS-CoV-2: severe acute respiratory syndrome coronavirus 2
SD: standard deviations
WBC: white blood cells
